# Women's autonomy, education and contraception use in Pakistan: a national study

**DOI:** 10.1186/1742-4755-2-8

**Published:** 2005-10-21

**Authors:** Shabana Saleem, Martin Bobak

**Affiliations:** 1 Reproductive Health Centre, Federal Government Services Hospital, Sector G\6, Islamabad Pakistan; 2 International Centre for Health and Society, Department of Epidemiology and Public Health, University College London, UK

## Abstract

**Background:**

It has been proposed that the autonomy of women is one of the mechanisms of how education influences contraceptive use in developing countries. We tested this hypothesis in a national sample of women in Pakistan.

**Methods:**

We used the 2000 Pakistan Reproductive Health and Family Planning Survey, which interviewed a national sample of ever married women aged 15–49 years (n = 6579). Women's decision autonomy was estimated from 9 questions on who makes decisions at home; movement autonomy was based on 6 questions on whether women need permission to visit places outside home. A number of socio-demographic variables were used in multivariate analysis to investigate the independent association between autonomy and lifetime and current contraception use and to assess the extent to which autonomy mediates the association between education and contraception use.

**Results:**

Decision autonomy was significantly associated with both lifetime and current contraception use; after controlling for covariates, the odds ratios for the highest vs. the lowest quintile were 1.8 (1.4–2.4) and 2.0 (1.4–2.8), respectively. Movement autonomy was not consistently associated with contraceptive use. Contraceptive use was strongly associated with women's education but this relation was not mediated by women's autonomy.

**Conclusion:**

Women's decision autonomy is significantly associated with contraceptive use but it does not appear to mediate the link between woman's education and contraception.

## Introduction

Family planning is an important issue for many developing countries worldwide, including South Asia. In Pakistan, despite a governmental programme supporting family planning and despite the improvements over the last few decades, total fertility rate remains high (4.8 in 2000) and current contraception use remains relatively low (20% in 2000) [1]. In 2004, Pakistan had lower contraception use than most other Muslim countries [2].

Fertility and contraceptive use in developing countries are associated with various markers of socioeconomic status, most prominent of which is women's education [3,4]; the well documented link between female education and use of contraception plays an important role in development of family planning policies in lower income countries.

In parts of South Asia, and elsewhere, women have a considerably lower social status and autonomy than men [4-7], and their low status and autonomy seems to be associated with lower fertility control [4,6,8]. Several reports showed a positive association between women's autonomy and contraception use [4,9,10]. Improving women's education has been seen one way to increase their status and autonomy [4,5,7,11], and it has been proposed that autonomy acts as a mediator of the link between education and contraception use [4,8,12].

This paper, using population data from Pakistan in 2000, has two objectives. First, to investigate the relation between women's autonomy and contraception use, and second, to assess the extent to which women's autonomy mediates the association between education and contraception use. The report is based on a secondary analysis of an existing dataset, and the choice of dimensions of women's autonomy was therefore restricted to what was available in the dataset. However, since both sets of questions (on movement and decision autonomy) consisted of very similar or identical questions that have previously been used, the results should be well comparable with previous studies.

## Methods

### The survey

We used data from the 2000 Pakistan Reproductive Health and Family Planning Survey (PRHFPS) [1]. The survey used a multi-stage sampling method to randomly select 7332 households (details have been described elsewhere [1]). In each selected household, ever-married women 15–49 years old were asked to participate in an interview. Interviews were conducted by specially selected and trained female interviewers between October 2000 and January 2001. The interview collected extensive information on household composition and socioeconomic circumstances and on women's socioeconomic, demographic, reproductive and family characteristics.

### Measurements

The basic demographic characteristics used in this analysis include province, urban/rural area of residence and women's age group and the number of living children. The standard of living of the household was characterised by the type of water supply, toilet facility and house construction. Women's education was taken as the basic measure of their socioeconomic status, and husband's education and employment in agriculture were taken as measures of husband's socioeconomic status (men employed in agriculture tend to have low income). The categorisation of these variables is shown in table 1.

**Table 1:** Numbers of women, contraceptive use (ever and current) and high (top quintile) decision and movement autonomy by socio-demographic characteristics.

	**Number (%)**	**Contraception ever (%)**	Contraception currently* (%)	**Decision autonomy %**	**Movement autonomy %**
Total sample	6579 (100)	40.2	28.0	20.0	21.4

Province					
Punjab	2895 (44.2)	44.8	31.5	24.7	26.6
Sindh	1791 (27.3)	32.7	24.0	15.8	12.7
NWFP	1167 (18.1)	43.3	26.4	19.5	15.6
Balochistan	606 (8.9)	29.9	21.6	6.8	29.2
Islamabad	120 (1.7)	64.0	48.3	43.3	41.7
*p-value*		*<0.001*	*<0.001*	*<0.001*	*<0.001*

Area					
Major urban	1524 (23.1)	62.0	47.3	30.4	34.1
Other urban	1302 (19.8)	46.7	30.4	19.3	22.6
Rural	3753 (57.1)	29.1	19.4	16.1	15.8
*p-value*		*<0.001*	*<0.001*	*<0.001*	*<0.001*

Married					
Yes	6361 (96.7)	40.8	28.1	18.5	20.4
No	218 (3.3)	23.4	23.4	63.8	50.0
*p-value*		*<0.001*	*0.127*	*<0.001*	*<0.001*

Age group					
<19	404 (6.1)	7.7	4.7	6.7	7.4
20–24	1081 (16.4)	23.7	13.8	11.2	10.9
25–29	1410 (21.4)	38.9	24.7	16.0	16.4
30–34	1233 (18.7)	48.7	33.4	21.9	20.9
35–39	1036 (15.8)	50.5	38.8	26.3	28.1
40+	1415 (21.5)	48.5	36.0	28.3	33.8
*p-value*		*<0.001*	*<0.001*	*<0.001*	*<0.001*

Water supply					
Piped	2310 (35.1)	53.7	38.7	23.7	25.1
Well in residence	3168 (48.2)	36.0	24.5	19.0	21.0
Other	110 (16.7)	24.3	15.9	15.2	14.6
*p-value*		*<0.001*	*<0.001*	*<0.001*	*<0.001*

House construction					
Katcha	2305 (35.0)	23.9	14.9	14.6	15.8
Semi-pacca	1673 (25.4)	40.7	26.7	20.8	20.1
Pacca	1895 (28.8)	55.5	41.1	23.5	26.9
Flat/house	490 (7.4)	64.4	49.0	32.0	34.3
Other	216 (3.3)	21.3	15.1	12.5	13.0
*p-value*		*<0.001*	*<0.001*	*<0.001*	*<0.001*

Toilet facility					
Flush	3258 (49.5)	54.4	39.6	24.7	26.2
Other in house	1047 (15.9)	33.1	20.5	14.1	15.1
No facility	2274 (34.6)	23.3	15.0	16.1	17.3
*p-value*		*<0.001*	*<0.001*	*<0.001*	*<0.001*

Education					
None	4604 (70.0)	32.7	22.1	17.8	18.3
1–5 yrs	804 (12.2)	48.8	34.2	21.4	23.3
6–10 yrs	801 (12.2)	63.7	47.0	25.6	31.6
11+ yrs	370 (5.6)	64.1	46.9	32.4	33.8
*p-value*		*<0.001*	*<0.001*	*<0.001*	*<0.001*

Living children					
None	869 (13.2)	2.1	0.6	12.2	14.5
1–2	1747 (26.6)	31.2	20.0	18.0	17.7
3–4	1884 (28.6)	50.2	34.6	24.2	24.4
5+	2079 (31.6)	54.7	40.3	21.2	24.6
*p-value*		*<0.001*	*<0.001*	*<0.001*	*<0.001*

Husband's education					
None	2561 (38.9)	28.7	19.2	20.7	21.5
1–5 yrs	1013 (15.4)	37.5	24.3	17.8	16.8
6–10 yrs	1929 (29.3)	48.4	34.2	19.7	21.1
11+ yrs	1076 (16.4)	55.5	41.2	21.0	25.9
*p-value*		*<0.001*	*<0.001*	*<0.001*	*<0.001*

Husband works in agriculture**					
No	5029 (79.0)	44.9	30.9	21.0	22.6
Yes	1338 (21.0)	25.3	17.9	9.3	12.4
*p-value*		*<0.001*	*<0.001*	*<0.001*	*<0.001*

* among non-pregnant women (n = 6372)

** among married women = 6361

Of the several dimensions of women's autonomy described in the literature [4,7], two were assessed in this study: decision autonomy and movement autonomy. Decision autonomy was estimated from 9 questions on decision making (e.g. children's health care, education, buying/selling property, what to cook etc) [1]. The responses were scored as follows: 2 points for decisions made by the woman; 1 point by decisions made jointly by both the woman and her husband; and 0 for all of decisions taken by others. We used the Cronbach's alpha coefficient to assess whether individual questions in the scale measured the same one underlying factor (the higher coefficient, the more internally consistent is the scale; values larger than 0.6 are considered acceptable). The Cronbach's alpha was 0.78, indicating a good internal consistency. The sum of valid (non-missing) responses was divided by the number of valid responses, resulting in the final score with values between 0 (no autonomy) and 1 (full autonomy).

The movement autonomy scale was based on 6 questions on whether permission by husband or a senior family member was required to go to several places (market, health centre, relatives' home etc)[1] The responses were scored as 2 (no permission required), 1 (depends) and 0 (permission always required). The Cronbach's alpha of these 6 sub-questions was 0.87, suggesting high internal consistency. The score was calculated identically as for decision autonomy, with the final score ranging from 0 (no autonomy) to 1 (full autonomy). For both scales, the questions were very similar to those used in previous studies; responses were not weighted.

An overall autonomy score, combining both dimensions, was also calculated. However, the correlation between the two individual scores (decision and movement) was relatively low (r = 0.35), and in exploratory analyses the overall score predicted contraception use less well than the individual scores, possibly because of combining two different dimensions of autonomy dilutes the effects of each scale. For these reasons, the overall score was not used in the final analyses.

The outcome of interest was contraceptive use. Women reported whether they ever or currently used any contraception, and they indicated the method they used. There were no differences in results between all and "modern" contraception methods (the latter excluded withdrawal and abstinence), and we therefore report the results on all types of contraception.

### Statistical analysis

**Figure 1:**
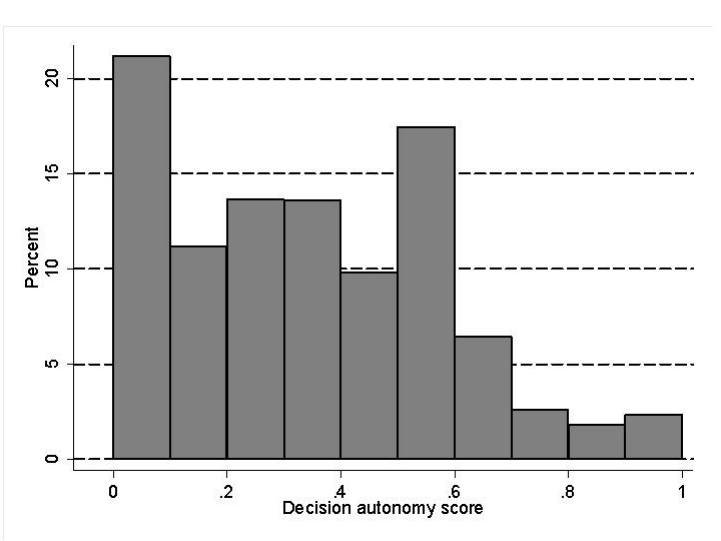
Distribution of the decision autonomy score.

**Figure 2:**
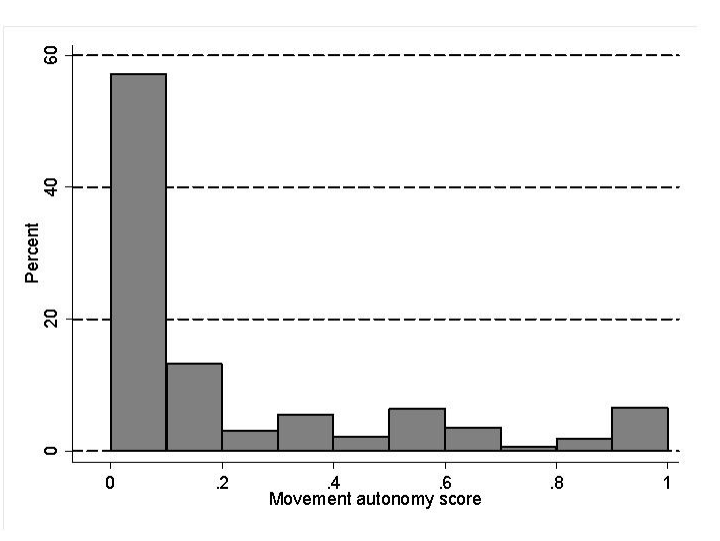
Distribution of the movement autonomy score.

The autonomy scores were distributed asymmetrically, with considerably more women with low autonomy than with high autonomy (figures 1 and 2). Women were therefore classified into quintiles of these three scores; since more than half of women reported no movement autonomy, there were only 4 categories.

Contraceptive use and autonomy were first tabulated by socio-demographic characteristics; statistical significance of the associations was assessed by chi square test. The odds ratios of contraceptive use (separately for ever and current use) by quintiles of decision and movement autonomy scores were estimated in logistic regression. (Since the data were collected in 367 primary sampling units, the sub-command "cluster" in STATA [13] was used to allow for potential autocorrelation within the sampling units). Second, the odds ratios were adjusted for the four basic demographic variables (province, urban/rural area of residence, age group and the number of living children). Third, the odds ratios were adjusted for all socio-demographic variables in table 1.

In the final step, we estimated the possible contribution of women's autonomy to the educational differences in contraceptive use. This was done by comparing the odds ratios by women's education before and after inclusion of women's autonomy into the following models: (i) crude (only education); (ii) adjusted for demographic factors (i.e. education plus province, urban/rural area of residence, age group and the number of living children); and (iii) fully adjusted (i.e. education plus all other socio-demographic variables listed in table 1). A reduction in odds ratio, after controlling for autonomy, would indicate a potential mediating role of autonomy. The reduction in odds ratios was quantified as [(odds ratio_(adjusted for autonomy) _– odds ratio_(not adjusted for autonomy)_) / (odds ratio_(not adjusted for autonomy) _-1)]. Since it is the difference in point estimates which matters, confidence intervals were not reported for this part of the analysis.

## Results

There were 6579 women with valid data (table 1). The life time prevalence of contraceptive use was 40%, and 28% of women were current users. There were marked and statistically significant differences in contraceptive use and autonomy scores by all socio-demographic characteristics. In exploratory multivariate analyses, most socio-demographic variables were associated with both indicators contraception use; the most prominent predictors of contraception use were the number of living children and women's education (not shown in table). For example, the fully adjusted odds ratio for the highest vs. the lowest educational category was 3.6 (2.5–5.0) for ever use and 2.4 (1.8–3.3) for current use. Distribution of both autonomy scores was highly asymmetrical, indicating low levels of autonomy of most women (figures 1 and 2).

Table 2 shows the association between the two types of autonomy with contraceptive use (ever and current, respectively). In crude analyses (column 2), decision autonomy was strongly associated with both lifetime and current contraceptive use; the odds ratios for the highest vs. the lowest quintile were 4.8 (3.8–6.0) and 5.0 (3.7–6.9), respectively. The higher odds ratios for current use, compared with ever use, is probably due to more recent and slightly more precise information on current use. Controlling for demographic variables reduced the odds ratios considerably (but further adjustment for all variables used in table 1 did not change the results). Nevertheless, in the full model, decision autonomy remained significantly associated with contraception use. The relation between movement autonomy and contraception use was considerably weaker than that of decision autonomy, and it was not linear. In the full model, the highest odds ratio was seen for the 4^th ^quintile.

**Table 2:** Odds ratios (95% confidence intervals) of contraceptive use ever by quintile of decision, movement and combined autonomy score.

*Type of autonomy *Quintile	Crude	Adjusted for demographic factors*	Fully adjusted**
**Contraception use ever**			

*Decision autonomy*			
1	1.0	1.0	1.0
2	2.16 (1.72–2.70)	1.49 (1.15–1.94)	1.39 (1.06–1.82)
3	2.98 (2.37–3.74)	1.59 (1.22–2.07)	1.42 (1.08–1.86)
4	4.88 (3.91–6.09)	2.12 (1.64–2.75)	1.81 (1.38–2.37)
5	4.82 (3.84–6.04)	1.88 (1.45–2.46)	1.82 (1.37–2.41)
p-value for trend	< 0.001	< 0.001	< 0.001

*Movement autonomy*			
1+2	1.0	1.0	1.0
3	0.97 (0.80–1.18)	1.23 (1.01–1.51)	1.25 (1.02–1.48)
4	1.43 (1.16–1.77)	1.51 (1.22–1.88)	1.54 (1.26–1.86)
5	1.72 (1.45–2.05)	1.14 (0.96–1.36)	1.13 (0.95–1.33)
p-value for trend	< 0.001	0.010	0.006

**Current contraceptive use**			

*Decision autonomy*			
1	1.0	1.0	1.0
2	2.36 (1.74–3.20)	1.82 (1.34–2.49)	1.73 (1.26–2.39)
3	3.16 (2.31–4.33)	1.88 (1.36–2.60)	1.73 (1.25–2.40)
4	5.34 (3.92–7.26)	2.54 (1.85–3.50)	2.23 (1.61–3.08)
5	5.04 (3.67–6.92)	2.15 (1.55–2.99)	2.01 (1.44–2.81)
p-value for trend	< 0.001	< 0.001	< 0.001

*Movement autonomy*			
1+2	1.0	1.0	1.0
3	0.92 (0.75–1.13)	1.13 (0.91–1.39)	1.13 (0.92–1.38)
4	1.36 (1.09–1.69)	1.29 (1.04–1.60)	1.29 (1.03–1.58)
5	1.66 (1.39–1.98)	1.06 (0.89–1.27)	1.03 (0.86–1.23)
p-value for trend	< 0.001	0.174	0.288

* adjusted for province, urban/rural area of residence, age group and the number of living children

** adjusted for province, urban/rural area of residence, age group, the number of living children, type of toilet, type of house, type of water supply, education, husband education and husband's occupation in agriculture.

Finally, we examined the potentially mediating effect of autonomy on the association between women's education and contraceptive use (table 3). We did so by adjusting the effect of education for autonomy and assessing the change in odds ratios. The changes in the odds ratios (between models without and with autonomy) in models adjusted for demographic and other covariates were modest, up to 11%. The small impact of adjustment for autonomy suggests that autonomy is not a major mediator (or confounder) of the link between education and contraception.

**Table 3:** Changes in the odds ratios of current contraceptive use by education after controlling for autonomy, at different levels of adjustment. Percentages in parentheses indicate reduction in odds ratios after adding autonomy into the model.

	Ever use				Current use			
	Without autonomy	Additionally adjusted for...			Without autonomy	Additionally adjusted for...		
				
		Decision autonomy	Movement autonomy	Both autonomy scores		Decision autonomy	Movement autonomy	Both autonomy scores

**Crude model **								

*Education** ***								
None	1.0	1.0	1.0	1.0	1.0	1.0	1.0	1.0
1–5 yrs	1.96	1.85 (-11%)	1.92 (-4%)	1.83 (-10%)	1.83	1.72 (-13%)	1.79 (-5%)	1.71 (-14%)
6–10 yrs	3.60	3.32 (-11%)	3.46 (-5%)	3.24 (-14%)	3.12	2.85 (-13%)	2.99 (-6%)	2.80 (-15%)
11+ yrs	3.66	3.09 (-23%)	3.52 (-5%)	3.04 (-23%)	3.10	2.59 (-24%)	2.98 (-6%)	2.57 (-25%)

Adjusted for demographic factors*								

*Education*								
None	1.0	1.0	1.0	1.0	1.0	1.0	1.0	1.0
1–5 yrs	2.25	2.21 (-3%)	2.27 (+2%)	2.20 (-4%)	1.93	1.89 (-4%)	1.94 (+1%)	1.90 (-3%)
6–10 yrs	4.21	4.14 (-2%)	4.22 (0%)	4.11 (-3%)	3.17	3.09 (-4%)	3.18 (0%)	3.11 (-3%)
11+ yrs	5.35	5.07 (-6%)	5.33 (0%)	5.03 (-7%)	3.69	3.47 (-8%)	3.69 (0%)	3.51 (-7%)

Fully adjusted**								

*Education*								
None	1.0	1.0	1.0	1.0	1.0	1.0	1.0	1.0
1–5 yrs	1.65	1.62 (-5%)	1.65 (0%)	1.60 (-8%)	1.43	1.40 (-8%)	1.43 (0%)	1.40 (-8%)
6–10 yrs	2.81	2.75 (-3%)	2.78 (-2%)	2.69 (-7%)	2.12	2.06 (-5%)	2.11 (-1%)	2.05 (-6%)
11+ yrs	3.56	3.35 (-8%)	3.51 (-2%)	3.27 (-11%)	2.41	2.27 (-10%)	2.40 (-1%)	2.26 (-11%)

* adjusted for province, urban/rural area of residence, age group and the number of living children

** adjusted for province, urban/rural area of residence, age group, the number of living children, type of toilet, type of house, type of water supply, education, husband education and husband occupation in agriculture.

## Discussion

In this secondary analysis of a large nationwide survey of women in Pakistan, we found that, in addition to a range of socio-demographic variables, women decision autonomy was significantly associated with contraceptive use. The data suggested, however, that autonomy did not mediate the association between women's education and contraceptive use.

The major strengths of the study are its representativeness, large sample size and comprehensive information on participating women and their household. The main weakness, on the other hand, is the cross-sectional design which may, in some aspects, obscure the temporality. For example, women's autonomy is partly derived from the number of living children, and the number of living children also influences contraception use; it is therefore difficult to clearly establish the temporality and causality of the effects. This problem, fortunately, does not affect the main focus of this paper. Education is a long-term and relatively stable characteristic, unlikely to be affected by the number of living children or autonomy. Similarly, it is unlikely that contraceptive use influences autonomy.

The second potential limitation is the opportunistic nature of these analyses, i.e. the fact that we relied on secondary data in defining the autonomy scores. Measurement of women's status is complex [7], with no general consensus on definition and most important autonomy dimensions. Using only two dimensions, from a number of those that have been suggested [4], is an oversimplification. However, similar or identical sets of questions have been used before and they appear to be useful to indicate women's autonomy in Pakistan and similar countries [9,10].

Our results confirmed the well known effects of most aspects of socioeconomic environment on contraceptive use (not reported in detail in this paper). From the various variables available, women's education had the most prominent role. This is consistent with most of the literature from South Asia and elsewhere [3,4,9,10].

The main focus of this analysis was on women's autonomy. Several findings deserve a note. First, the distribution of both autonomy scores was skewed towards low autonomy levels (figures 1 and 2). It has been pointed out that the western view, seeing low autonomy as negative, is not necessarily correct [7,14]. However, if women's decision authority is indeed associated with fertility and other health related characteristics, as our and others' results suggest, then the low levels of decision autonomy are of concern.

Second, decision autonomy remained significantly associated with contraceptive use, even after controlling for a battery of socio-demographic variables. The fully adjusted effects were not huge but they still indicate an approximately two-fold difference in contraceptive use between women with least and most autonomy. This is not negligible. By contrast, movement autonomy was not associated with contraceptive use after adjustment for other variables. This is consistent with a recent study of Pakistan which found only a limited role of women's mobility and uptake of reproductive health services [14].

Finally, only a few studies investigated the mediating effect of autonomy (on the influence of education) in individual level data, with conflicting results. While in Bangladesh women's autonomy played a major role [12], analysis of Indian data found that autonomy did not mediate the link between education and contraception [15]. In our data, both autonomy scores were associated with women's education (and most other socio-demographic characteristics) but they did not appear to mediate the effect of women's education on contraceptive use. High women's autonomy generally is generally seen as desirable (although its significance may be different in different settings [7,14]); however, our findings suggest that the impact of women's education on contraceptive use is independent from either decision or movement autonomy.

## Competing interests

The author(s) declare that they have no competing interests.

## Authors' contributions

SS and MB jointly developed the principal idea for the analyses. SS obtained and analysed the data, reviewed the literature and commented on the draft. MB supervised the analyses and drafted the paper.
